# Grand Challenge in Veterinary Imaging: Nothing Is More Constant Than Change

**DOI:** 10.3389/fvets.2022.936754

**Published:** 2022-06-24

**Authors:** Sibylle Kneissl

**Affiliations:** Diagnostic Imaging, Clinic for Small Animals and Horses, University of Veterinary Medicine, Vienna, Austria

**Keywords:** veterinary imaging, diagnostic imaging, bioimaging, teleradiology, workplace

## Introduction

Veterinary imaging has been challenged by rapid technological advances. Analogous to the Heraclitus of Ephesus maxim quoted in the title, my discussions with colleagues have led me to conclude that we witness constant change. Evidence for this can be seen in the variety of subject names used in veterinary imaging, in the accepted core fields for the subject, and in the workplace, meaning this community identity and these values are now the norm. We are not alone in facing challenges of this type. Experiences in other domains can be illuminating for ours. The threshold concept of Meyer and Land (1) was developed to describe learning and teaching in universities. Threshold concepts have characteristic properties and knowledge of them can help with understanding the responses of the veterinary imaging community to the changes or transitions we see in our technologies and modalities. They describe in their domain how threshold concepts can be seen in education in transitions between core subjects (e.g., imaging, surgery, or internal medicine) and non-core subjects (e.g., physics, chemistry, or informatics). Being threshold concepts implies that these transitions can be transformative, irreversible, integrative, bounded, and potentially troublesome. These terms might equally apply to transitions in veterinary imaging. Being transformative is understood as having the potential for a significant shift in understanding. Transformations may be irreversible in that a new way of thinking might be difficult to reconcile with thinking based on a prior understanding. The integrative concept supports the idea of Lave and Wenger (2), who proposed that learners are more likely to progress when actively engaged in communities of practice. Hence for our domain, senior radiologists or other health professionals can enhance newer learners' participation and sometimes may learn from the newcomer. Often, but not always, a threshold may have bounded features. Boundedness may lead to quietly dropped and not understood areas in a subject and might explain academic and clinical specialty territories. Threshold concepts can be potentially troublesome if new knowledge is ritual, inert, difficult, alien, tacit, or simply provided in a foreign language (1). Thus, the concept of thresholds is rich with potential. Thresholds between core and non-core fields have the power to unite or separate individuals from rapidly growing knowledge or communities.

The viewpoint of this article is from a privileged distance and describes how veterinary radiologists perceive or experience their core and non-core subjects. This article also outlines risks and offers solutions on how inter-professional teams can work together to design the future of veterinary imaging.

## Change In Subject Name and Shift of Core Fields to Other Subjects

In the 1950s, veterinary practitioners, who were interested in learning more about radiology and contributing new knowledge to the discipline, met and collated their knowledge in the *Journal of Veterinary Radiology*. Thrall (3) expanded the scope of the Journal as the subject itself expanded, leading eventually to a decision to append “& *Ultrasound*” to the Journal title in 1991. In 2015, Frontiers implemented a new, inter-disciplinary journal section called veterinary imaging, setting a new horizon for the field and implementing intelligent algorithms to smooth the editorial and review process (4). The scope of the journal is set up for many clinical specialties such as internal medicine, surgery, neurology, oncology, and pathology, as well as non-clinical fields such as physical and engineering sciences. At about the same time, various European academic disciplines that use imaging in the workplace, met and reorganized themselves as *BioImaging* collaborators with the goals of reaching a critical mass to be internationally recognized and centralizing expertise with open access to high-end imaging modalities. The corresponding *Correlated Multimodal Imaging Nodes in Life Sciences* was mainly triggered by international funding such as from the European Cooperation in Science and Technology (5). Besides the transformation from veterinary radiology to imaging, emerging subject terms are radiomics (6), veterinary informatics (7), radiogenomics (8), and theranostics (9, 10). These terms signal the shift from intra-disciplinary to inter-disciplinary teams, where researchers collaborate in common working areas, such as radiology and information technology aim at extracting imaging patterns paired with subsequent automatic analyses (radiomics, veterinary informatics), radiology and genomic phenotyping (radiogenomics), radiology and targeted pharmacotherapy (theranostics) for dedicated radiotracers (11), or with images obtained using specific contrast media (12). An obvious threat to these dynamics for veterinary imaging is being absorbed by other disciplines. This threat is fostered by the paucity of funding opportunities for research in veterinary imaging (13–15) and illustrated in the publication of guidelines for magnetic resonance imaging without a specialized radiologist as a co-author (16). The future of veterinary imaging has already begun in the shape of inter-disciplinary teams, where new knowledge is created in the friction zone of various disciplines. In this melting pot, it is our responsibility to develop collective competence (17). Collective or team competence does not simply correlate with the sum of all individual competencies, it is more a network of complex interactions among individuals. Collective competence is dynamic and strongly depends on the context (18). A good analogy for collective competence is the swarming behavior seen birds where adherence to a simple algorithm (watching the seven closest neighbors) can result in, a useful and functional group effect (19, 20). One way forward to create collective competence is to integrate emotions into the workplace (21, 22). Addressing feelings such as face-to-face interactions, managing other people's feelings and one's own, and expressing emotions such as smiling is crucial for successful teamwork (18). Another way forward is exploring situated shared competence in healthcare (23).

## Change In the Workplace Including a New Setting for Teaching and Training

Veterinary teleradiology was introduced in the 1990s (24). With increasing internet speed, cloud computing, and established private companies veterinary teleradiology is used worldwide for primary interpretations, on-call coverage, and second opinion consultations (25). Apart from continuous challenges such as licensing and credentialing, regulations, technology, and staffing models (26), the advantages of the remote workplace in veterinary imaging mean there is no commuting requirement, and more office hours are associated with more income, and wide accessibility to high-level expertise for clinical decision making. The 2019 coronavirus disease pandemic has smoothened the last technical hurdles, such as audio and visual signal transfer from home offices to clinics or access to sonographic monitors, where the transducer is moved by a novice but under expert guidance. However, there are risks associated with teleradiology such as it might prove too inconvenient for a remote radiologist to obtain additional images or collateral information. The reasons for success of emergency teleradiology is largely due to the limited number of indications, little need to review prior examination results, and the limited amount of collateral information needed (27). Currently, there is a tendency in health checks to simply push images to remote readers with a lack of relevant corresponding major findings, meaning the quality of outsourced teleradiology might become less than optimal. The other risk associated with teleradiology is that of losing academic staff who are driven away by academic workload (28) and salary, resulting in higher service and teaching loads for the remaining academic staff members, with reduced capacity to do research (29) and in turn to develop the field of veterinary imaging. Especially during the pandemic, high psychosocial work demands decrease the wellbeing of veterinary academia (30), and this situation might be prolonged in less well-staffed academic institutions. A solution is to provide new contract forms such as part time or remote work possibilities for highly qualified young radiologists. Further post-graduate training packages or additional variable salaries as well as dual career opportunities (for the recruited person and their partner), living support, or flexible working hours could compensate for otherwise less attractive perceived academic workload in all academic disciplines.

## Change In Community and Values

As mentioned above, inter-disciplinary teams are emerging. Apart from this, imaging teams are challenged by the problems of retaining an adequate number of permanent, board-certified, and onsite workers to run the service of diagnostic imaging and guarantee supervised training.

Communities of trainers and trainees are active on different communication platforms: trainers on websites communicating with email, and trainees, mainly generation Y (born 1981–1995) using social media. Trainers and trainees are likely integrated into different extra-organizational communities such as congress or exam communities for the former, vs. TikTok or Instagram for the latter. Opportunities regarding these different communities include digital literacy for attendees, networking, or offsite (non) formal training. Risks are intra-organizational team separation or unequal loading of boarded vs. non-boarded trainers. Too high demands and unfair rewards are drivers against the engagement of boarded trainers (31, 32). Regarding Austrian veterinary imaging specialists in training, my personal experience is that their main interests lie outside of scientific writing; most of these students have integrated (autonomous) extrinsic motivation if they can find supportive conditions. For example, their scientific project is residency-related and that is why they engage. Since they do not find many other supportive conditions (e.g., additional study or research time) and publishing has become more cumbersome, they are not highly motivated to continue scientific projects after having finished their residency. Again, in Austria, academic newcomers, highly qualified radiologists, stop their career in the post-doctoral phase to convert to a career with fewer hurdles and more income; thus, it is hard to encourage upcoming young European academics to stay in the field. This may be a European phenomenon and less applicable in Asia or elsewhere, but I doubt this is so. A solution has been for resident recruitment is to invest in candidates who are most likely to stay in academia, develop a good feedback culture, solve problems before candidates drop off, offer transparent careers with attractive remuneration packages, and foster inter-disciplinary teams for high-quality studies that motivate young researchers to stay in the field. Currently, many of us manage the best trained generation with outdated “carrot and stick” approaches. This is an error as these individuals expect and respond best to motivations that arise from interesting tasks, responsibility, and compelling personal development opportunities (Motivation 2.0). These approaches encourage high level performance (33).

## Conclusion

We do not know the future of veterinary imaging, but it will likely be highly dynamic. The imaging market is likely to grow (34) and there will be more imaging machines than trained radiologists, so teleradiology together with machine-based learning will continue to grow. Teleradiology companies attract highly qualified young radiologists for the opportunity to work part-time and gain relatively more money. Hence, it is likely that veterinary imaging will be partly replaced by machine learning algorithms and partly integrated into other disciplines as is already the case in cardiology or dentistry. Current academic leaders must offer more transparent careers with attractive part-time positions and remuneration packages soon. This generation is highly qualified and high-performing. If they find adequate content factors such as interesting tasks or personal development opportunities, they will engage with the full spectrum of veterinary imaging.

## Author Contributions

SK contributed to conception, design of the study, and wrote revised and approved the submitted version.

## Conflict of Interest

The author declares that the research was conducted in the absence of any commercial or financial relationships that could be construed as a potential conflict of interest.

## Publisher's Note

All claims expressed in this article are solely those of the authors and do not necessarily represent those of their affiliated organizations, or those of the publisher, the editors and the reviewers. Any product that may be evaluated in this article, or claim that may be made by its manufacturer, is not guaranteed or endorsed by the publisher.
